# Mechanisms Underlying the Action and Synergism of Trastuzumab and Pertuzumab in Targeting HER2-Positive Breast Cancer

**DOI:** 10.3390/cancers10100342

**Published:** 2018-09-20

**Authors:** Babak Nami, Hamid Maadi, Zhixiang Wang

**Affiliations:** Signal Transduction Research Group, Department of Medical Genetics, Faculty of Medicine and Dentistry, University of Alberta, Edmonton, AB T6G 2H7, Canada; namimoll@ualberta.ca (B.N.); hmaadi@ualberta.ca (H.M.)

**Keywords:** trastuzumab, pertuzumab, breast cancer, HER2, HER family receptors, mechanisms, synergism

## Abstract

Human epidermal growth factor receptor (HER) 2 (HER2) is overexpressed in 20–30% of breast cancers. HER2 is a preferred target for treating HER2-positive breast cancer. Trastuzumab and pertuzumab are two HER2-targeted monoclonal antibodies approved by the Food and Drug Administration (FDA) to use as adjuvant therapy in combination with docetaxel to treat metastatic HER2-positive breast cancer. Adding the monoclonal antibodies to treatment regimen has changed the paradigm for treatment of HER2-positive breast cancer. Despite improving outcomes, the percentage of the patients who benefit from the treatment is still low. Continued research and development of novel agents and strategies of drug combinations is needed. A thorough understanding of the molecular mechanisms underlying the action and synergism of trastuzumab and pertuzumab is essential for moving forward to achieve high efficacy in treating HER2-positive breast cancer. This review examined and analyzed findings and hypotheses regarding the action and synergism of trastuzumab and pertuzumab and proposed a model of synergism based on available information.

## 1. Introduction

There are four members in the HER receptor family. They are epidermal growth factor (EGF) receptor (EGFR/HER1/ErbB1, HER2/ErbB2, HER3/ErbB3, and HER4/ErbB4 [1,2]. All HER receptors, except for HER4, have been implicated in breast cancer [3,4,5,6,7,8,9,10,11,12]. EGFR, HER2 and HER3 are overexpressed in 30–40%, 20–30% and ~20% of breast cancer cases, respectively [4,11,13,14,15,16,17]. Targeting HER2 in treating HER2-positive breast cancer has proven to be an effective therapeutic strategy [18,19]. Since approval by the FDA in 1998, trastuzumab, a HER2 antibody, has changed the paradigm for treating HER2-positive breast cancer [19,20]. However, acquired trastuzumab resistance has developed with time, which needs to be overcome [19,21,22]. Pertuzumab is another HER2-targeting antibody. Its recent approval by the FDA to be used in combination with trastuzumab and docetaxel has significantly improved the outcome of the patients with metastatic HER2-positive breast cancer [11,19,23,24,25]. It has been shown recently that adding pertuzumab to adjuvant trastuzumab and chemotherapy significantly improves the outcomes among patients with HER2-positive early breast cancer [26]. However, the lack of a good understanding of the mechanisms underlying the action and synergism of trastuzumab and pertuzumab severely limit its application and efficacy. Indeed, despite these achievements, the persisting high toll of deaths necessitates newer therapies and combinations [27]. In this review, we will focus on the reported mechanisms and hypotheses underlying the action and synergism of trastuzumab and pertuzumab in targeting HER2-positive breast cancer.

## 2. The Roles of HER Receptors in Breast Cancers

Breast cancers are classified as five intrinsic subtypes based on their gene expression profiles revealed by microarray: luminal-like subtypes A and B (expression of hormone receptors and luminal cytokeratins 8 and 18), basal-like (also called triple-negative breast cancer (TNBC), typically with no expression of estrogen receptors (ER), progestin receptors, and HER2), HER2-positive (HER2+), and normal-like [28,29]. HER receptors have been implicated in the development of many types of human cancers, especially breast cancer. Overactivation of HER receptors is mostly due to overexpression driven by gene amplification, but also could be due to the truncation of the extracellular domain, mutation in kinase domain, or co-expression of HER receptor ligands. The overactivation of HER receptors drives cancer development [5,30,31].

Overexpression of EGFR is observed in 20–30% of breast carcinoma. While high percentage of HER2-positive breast cancer cells also overexpress EGFR, approximately 50% of TNBC cells overexpress EGFR [12,30,32]. Overexpression of EGFR has been frequently associated with large tumor size and poor clinical outcomes [30,32,33,34].

ErbB2 overexpression occurs in 20–30% of breast cancers and ovarian cancers [11,13,14,15,16,30,35]. HER2 mutations are observed in approximately 1.6% of breast cancer patients [36]. Patients whose breast tumors overexpress the ErbB2 have a significantly lower survival rate and a shorter period before relapse than patients without ErbB2 overexpression [13,35,37]. Moreover, ErbB2 overexpression has been positively correlated with lymph node metastasis in breast cancers [38,39]. Overexpression of HER2 also increases tumorigenicity in nude mouse xenograft models [40,41,42]. ErbB receptors have been the top choice for breast cancer therapies [6,8,16,43].

Overexpression of ErbB3 occurs in about 20% of breast cancers [15]. Overexpression is mostly due to increased transcription [15,44]. Overexpression of HER3 alone does not promote anchorage-independent growth; however, when expressed together with HER2, HER3 strongly stimulates cell growth [30,45]. Different from other HER receptors, both oncogenic and tumor-suppressor functions have been reported for HER4 [46,47,48,49].

## 3. HER Receptors and Cell Signaling

**Figure 1:**
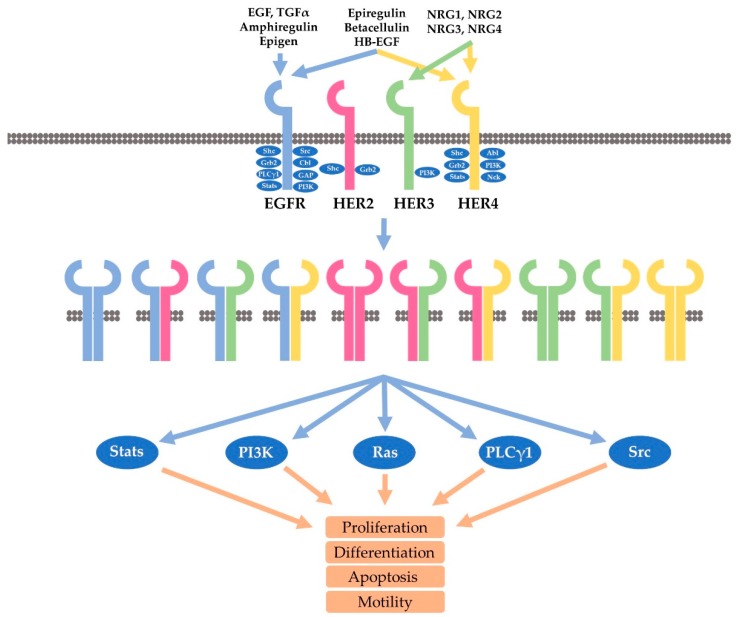
The activation of HER receptors and the downstream signaling cascades. Four members of HER receptors interact with 11 ligands, which results in the formation and the activation of 10 different homo- and heterodimers. Activated HER receptors promote many signaling cascades affecting many key biological outcomes.

There are more than 60 receptor tyrosine kinases (RTKs) that have been identified in the human genome [50]. Like other RTKs, HER receptors are single transmembrane proteins which have an N-terminal extracellular domain, a transmembrane helix, and a cytoplasmic domain [51]. The extracellular domain contains four subdomains, including the ligand binding subdomains (domain I and III), and receptor dimerization subdomains (domain II and IV). The intracellular domain is composed of a tyrosine kinase domain and a C-terminal regulatory domain (Figure 1) [52].

EGFR, a 170 kD single polypeptide chain, is the prototype of the HER family receptor [53,54,55,56,57,58]. While EGFR and HER4 are fully functional RTKs capable of signaling both as a homo- and heterodimers following the binding to various ligands, the other two members, including HER2 and HER3, are different. HER2 is an orphan receptor without a ligand and HER3 is a lack of kinase activity. However, through ligand-induced heterodimerization, all HER receptors could be fully activated to mediate cell signaling [2,16,59,60,61,62,63,64,65,66,67,68,69,70,71,72,73].

Besides EGF, another ten ligands have been identified to bind to and stimulate HER receptors. These ligands form the EGF family of peptide growth factors and are subdivided into three groups based on their binding partners (Figure 1). EGF, epigen (EPG), amphiregulin (AR), and transforming growth factor (TGF) form one group that specifically binds to EGFR. HB-EGF, epiregulin (EPR), and betacellulin (BTC) form the second group that binds to both EGFR and HER4. The four neurogulins, including NRG1, NRG2, NRG3, and NRG4, form the third group that binds to HER4. However, NRG and NRG2 also bind to HER3 (Figure 1) [5,30,31,74,75,76]. Through the distinctive binding specificity and affinity, each ligand contributes in a unique manner to regulate the activation and signaling of the four HER receptors [2].

Our understanding of HER receptor dimerization has been greatly enhanced due to the determination of the structures of the HER receptor extracellular domains. So far, the structures of all HER receptors without ligand have been determined. In addition, the structures of ligand-bound EGFR and HER4 have also been determined. Many structures of HER receptors binding to antibodies or antibody mimics have also been revealed [51,77,78]. With the support of other evidence, a comprehensive picture regarding ligand-receptor interaction and HER receptor dimerization have emerged. In total, ten different homo- and heterodimers are formed by four HER receptors (Figure 1) [1,79].

Structure studies indicate that the conformations of the receptors can only exist in two forms: a tethered form and an extended form. In the tethered form the receptor is unable to dimerize due to the buried dimerization element. However, in the extended form the dimerization elements of the receptor are fully exposed to allow the receptor dimerization. It has been demonstrated that the rigid nature of the receptor extracellular domains restricted or “clicked” the receptors only to these two forms [51,78].

In is significant and interesting to find that HER2 extracellular domains are already in extended form in the absence of ligands. The subdomains I and III of HER2 extracellular domain interact directly to stabilize HER2 to the extended form. The close interaction between subdomain I and III leaves no space for a ligand in between, Therefor, HER2 is an orphan receptor by nature [79]. Thus, HER2 maintains a ligand-independent and constitutively activated conformation. Indeed, HER2 spontaneously forms homodimers when overexpressed in cells, and all the other HER receptors dimerize preferably with HER2 [1,30]. Moreover, the overexpression of HER2 (but not the other HER receptors) transforms cells, and HER2 overexpression is associated with poor prognosis in breast cancer [79].

On the other hand, HER3 homodimer is generally believed as non-functional due to lack of kinase activity. However, HER3 possesses very low kinase activity (1/1000th kinase activity of EGFR) and thus it is still possible that HER3 homodimers may be functional [30].

Through homo- or heterodimerization, all HER receptors are activated, which induces the phosphorylation of multiple tyrosine residues in the C-terminal regulatory region. Various studies including large-scale phosphoproteomic screening has identified more than 100 proteins that potentially bind to HER receptors (Figure 1) [12,30,80,81,82,83,84,85,86,87,88]. Several interesting features are revealed through the mapping of these tyrosine phosphorylation residues. Both EGFR and HER4 bind to many different downstream proteins. EGFR binds to Grb2, Shc, Src, PLC-γ1, Crk, Stat5, Ptp-2c, and SHP1. HER4 binds to Syk, RasA1, Abl, Crk and Vav2, and Grb2. However, the signaling pathways linked to HER2 and HER3 are very specific and limited. HER3 contains multiple phosphor tyrosine residues that bind to p85, and as such, HER3 strongly activate the PI3K-Akt pathway. On the other hand, HER2 is mostly engaged in Shc/Grb2-mediated activation of Erk pathways. Due to the distinctive binding to various downstream signaling proteins, the heterodimerization of HER receptors allows the activation of more signaling cascades than the homodimer of HER receptors. Based on the binding specificity, it is likely that the HER2 homodimer will mostly stimulate the activation of the Ras-ERK pathway, and the HER2-EGFR heterodimer may function similarly to EGFR homodimer. However, the HER2-HER3 heterodimer could be much more powerful than either the HER2 homodimer or HER3 homodimer because the HER2-HER3 heterodimer could fully activate all available HER2 and HER3 receptors, and the HER2-HER3 heterodimer could strongly activate the PI3K-Akt pathway in addition to the Ras-ERK pathway. Indeed, much research has linked the PI3K-Akt pathway to HER2-HER3 signaling and to HER2-positive breast cancer.

PI3K could be activated by HER receptors either directly through the interaction between its p85α subunit and HER receptor or indirectly through the activated Ras [89,90]. A negative regulator of PI3K is the phosphatase and tensin homologue deleted on chromosome 10 (PTEN). The function of PI3K in cell survival is mediated by Akt, a serine threonine (Thr) kinase [91,92,93]. Akt contains an N-terminal Pleckstrin homology (PH) domain, a C-terminal regulatory domain, and a central kinase domain. Akt is recruited to the plasma membrane by the interaction of its SH3 domain with PIP3 (generated by PI3K), which induces the conformational change of Akt to allow the phosphorylation of its Thr 308 by membrane-localized 3-phosphoinositide-dependent kinase 1 (PDK-1). Following the additional phosphorylation of Ser 473 by rapamycin complex 2 (mTORC2), Akt is fully activated. Akt controls various cellular functions by phosphorylating several intracellular proteins, including the glycogen synthase kinase 3 (GSK3), the BCL2-associated agonist of cell death (BAD), and forkhead box O transcription factors (FoxO). Akt also activates mTORC1, which protects the cell from undergoing apoptosis [92,93,94].

## 4. Mechanisms Underlying the Action of Trastuzumab

HER2 is an attractive therapeutic target for the treatment of HER2-positive breast cancer [6,8]. As a humanized recombinant monoclonal antibody to HER2, trastuzumab binds to HER2 domain IV that is close to the HER2 juxtamembrane region. Trastuzumab is the first HER2-targeted therapy that was approved by the FDA for the treatment of metastatic breast cancer. It selectively exerts antitumor effects in HER2-positive breast cancer patients [6,8].

**Figure 2:**
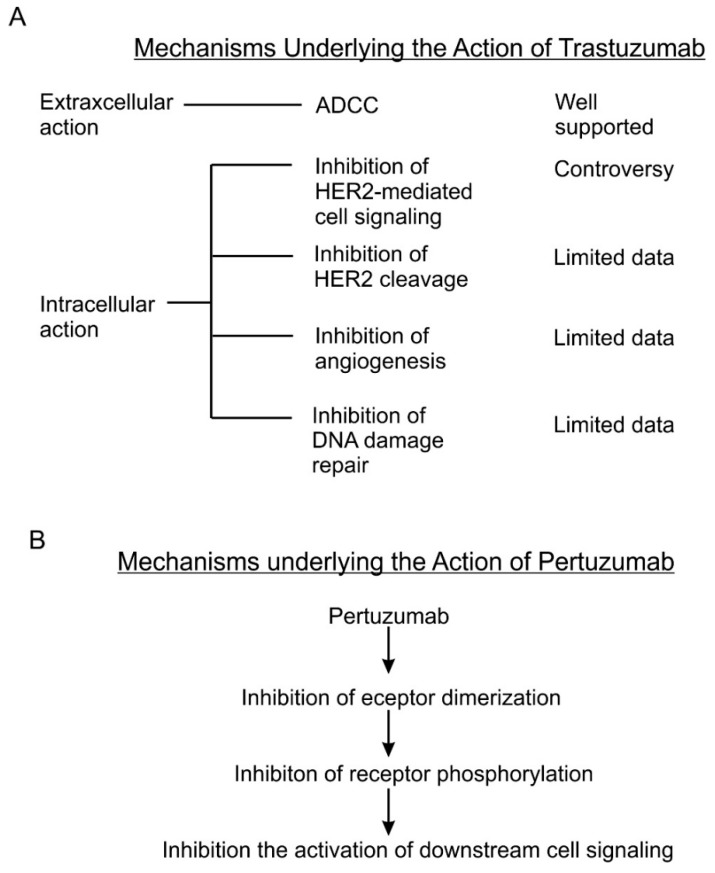
The potential mode of action of trastuzumab and pertuzumab. (**A**) Five mechanism have been proposed and partially tested regarding the action of trastuzumab. (**B**) The mechanism of pertuzumab is likely through the inhibition of HER2 dimerization and activation.

Although many mechanisms have been proposed for its antitumor activity, the exact mechanisms remain unknown. As summarized by several earlier reviews [6,8,95], several mechanisms including both intracellular and extracellular mechanisms are proposed for the action of trastuzumab (Figure 2A).

Antibody-dependent cell-mediated cytotoxicity (ADCC) is identified as the extracellular action of trastuzumab. The Fc receptor on immune effector cells, principally natural-killer (NK) cells, recognizes the Fc portion of trastuzumab in the targeted cancer cell and attacks the cancer cells. Action through ADCC has been supported by much evidence as a major mechanism of trastuzumab [96,97,98,99,100,101,102,103,104]. This mechanism is also supported by recent studies [101,104]. Recent studies in this area have focused on how to enhance the ADCC mediated by trastuzumab. It is reported that trastuzumab-induced ADCC could by augmented by enhancing NK cell activities [105,106], by modifying the antibody itself [107,108,109], and by inhibitors to various proteins including caspase [110], CD112R and TIGIT [111], Histone deacetylase (HDAC), and Adams [112]. Moreover, chemotherapeutic drug including Tanxanes [113] and tyrosine kinase inhibitors [114,115] are also found to enhance trastuzumab-mediated ADCC.

Many intracellular mechanisms have been proposed for trastuzumab action; however, the data are controversial [83]. The proposed intracellular mechanisms include: (1) inhibition of intracellular signal transduction leading to cell proliferation. While this mechanism has been the basis for developing trastuzumab and has been referred by most reviews [6,116,117], this mechanism is not supported by experimental data. Most experimental data indicate that trastuzumab does not inhibit, but under certain conditions actually stimulates HER2 phosphorylation [6,8,95,118,119]. The data regarding the effects of trastuzumab on HER2 dimerization, HER2-mediated activation of Akt, Erk and other signaling proteins [120,121,122,123], and HER2 endocytosis/downregulation [120,121,122,123] are all controversial. Some data suggest that trastuzumab may affect cell signaling and cell cycle progression by regulating gene expression through an undefined mechanism [118,124]. (2) Inhibition of the proteolytic cleavage of HER2 extracellular domain. p95HER2 fragments are truncated HER2 proteins characterized by the lack of extracellular domain (ECD), but still possessing tyrosine kinase activity [125,126,127,128,129]. p95HER2 fragments arise by two different mechanisms: (i) proteolytic shedding/cleavage of p185HER2 by zinc-containing metalloproteinase, including A disintegrin and metalloproteinases (ADAM) and matrix metalloproteinase (MMP) family members [130,131,132]; and (ii) Alternative initiation of translation. p95HER2 could be generated by alternative initiation of translation from methionines located near the transmembrane domain of the full-length molecule [133]. Breast cancer patients expressing p95HER2 are more likely to develop nodal metastasis [134] and have worse prognoses than those predominantly expressing the full-length receptor [135]. It was reported that trastuzumab blocked both basal and induced cleavage of p95HER2 [126]. (3) Inhibition of DNA repair. Chemo- and radiotherapies induce DNA damage in treated cancer cells and cancer cells may minimize this damage by repairing the damaged DNA. Some early studies suggest that trastuzumab partially blocks the repairing of damaged DNA [136,137,138]. However, all these data were published in the 1990s from one research group. (4) Inhibition of angiogenesis. Cancer cells promote angiogenesis to support tumor growth. Trastuzumab was shown in a preclinical murine xenograft tumor model to inhibit the angiogenesis [139,140].

## 5. Mechanisms Underlying Trastuzumab Resistance

Since its introduction in 1999, trastuzumab has changed the paradigm of treating metastatic HER2-positive breast cancer patients. While it significantly improved the treatment, the resistance, both innate and acquired, has posed big challenge [141,142]. The overall response rate is about 50% with a significant percentage (approximately 40%) of metastatic patients demonstrating primary resistance. Moreover, most of the patients who initially responded to trastuzumab treatment quickly acquired resistance. Scientific communities have been studying the mechanisms underlying the resistance in the hope of overcoming this it [142,143,144].

Many resistance mechanisms have been identified. The resistance may arise due the altered HER2 expression status of the cancer cells [145,146]. The resistance may also arise due the alteration of HER2 molecule structures, such as proteolytic truncation of HER2 extracellular domain, which prevents the binding of trastuzumab to the truncated but constitutively activated HER2 [127,128,147,148]. Activation of other HER receptors such as EGFR, which compensate the lost HER2 signaling due to trastuzumab inhibition [149,150], or activation of HER2 through a mechanism that is not sensitive to trastuzumab [151,152]. Constitutive activation of downstream signaling pathways due to mutations are also a major mechanism for trastuzumab resistance. The most prominent case is the constitutive activation of PI3K-Akt-mTor pathway due to gain of function mutation of PI3K, and the loss of function of PTEN [153,154,155,156,157,158,159]. Some other mechanisms are also reported, including FCγ receptor polymorphism [160,161], miRNAs [162,163], and Mucin 4 expression induced by TNFα [164].

As HER2/HER3 heterodimer-mediated activation of PI3K-Akt-mTor has been considered the most important signaling pathway driving the development of breast cancer, and constitutive activation of this pathway identified as a major resistant mechanisms for trastuzumab resistance, combined inhibition of both HER2 and PI3K-Akt-mTor has been explored to overcome trastuzumab resistance [165,166]. Most research has demonstrated that additional inhibition of PI3K-Akt-mTor could overcome trastuzumab resistance in HER2-positive breast cancers [156,167,168,169,170].

## 6. Mechanisms Underlying the Action of Pertuzumab

As a fully humanized recombinant monoclonal antibody, pertuzumab represents a new class of agents that inhibit HER2 dimerization [19] (Figure 2B). Pertuzumab specifically interacts with the subdomain II of HER2 extracellular domain, sterically blocking a binding pocket necessary for receptor dimerization, thus blocking HER2 dimerization mediated by the HER2 dimerization domain [77]. Indeed, the same research showed that pertuzumab blocked heregulin-induced heterodimerization between HER2 and HER3 [77]. Inhibition of dimerization will lead to the blocking of HER2 activation and HER2-mediated downstream signaling [19]. This understanding is mostly based on important early research [171]. This research showed that pertuzumab blocks the association of HER2 and HER3, diminishes ligand-activated HER2 signaling including Erk activation, and inhibits the growth of human breast cancer cell lines only in the presence of ligand (heregulin) [171]. This research was conducted with breast cancer cell lines that co-express both HER2 and HER3 in the context of heregulin stimulation. Subsequent brief research suggests the synergistic effect of trastuzumab and pertuzumab on breast cancer survival, but showed that pertuzumab alone is less effective in blocking Akt phosphorylation than trastuzumab and both antibodies have no effect on Erk phosphorylation in BT474 cells [172]. It was further reported that pertuzumab disrupts EGF-induced heterodimerization of HER2 and EGFR in ovarian cancer cells, expressing both EGFR and HER2. Pertuzumab also inhibits in vitro and in vivo growth of the same ovarian cancers [173]. Moreover, pertuzumab can abrogate the inhibitory effect of HER2 on the degradation of HER3 [174]. A recent study showed that both trastuzumab and pertuzumab inhibit NRF2 function in ovarian cancers and the combination of the antibodies produces more potent effects than a single antibody alone [175]. In summary, while the data regarding the mode of action of pertuzumab is quite limited, the available data mostly support the role of pertuzumab in blocking the heterodimerization of HER2, which in turn blocks the activation of HER2- and HER3-mediated signal transduction pathways leading to cancer cell proliferation and survival.

Pertuzumab was approved by the FDA in 2012 to be used in combination with trastuzumab and docetaxel for treating metastatic breast cancer patients. This approval is based on the clinic trial results reported the same year by the CLEOPATRA Study Group [167]. This treatment regime has significantly improved the outcome of the patients with metastatic HER2-positive breast cancer and is now the standard first-line treatment for HER2-positive metastatic breast cancer [11,19,23,24,25,176]. It has also recently been shown that adding pertuzumab to adjuvant trastuzumab and chemotherapy results in better outcomes among patients with HER2-positive early breast cancer [26]. As pertuzumab has only been introduced for a short period, there is not enough data and research regarding the resistance. However, the persistent high death toll and lower response rate among patients previously treated with trastuzumab suggests the presence of resistance and poses challenges [27,177].

## 7. The Mechanisms Underlying the Synergism of Trastuzumab and Pertuzumab

While a better outcome is achieved by the combination of pertuzumab and trastuzumab, very little is known regarding the mechanisms underlying the synergism of trastuzumab and pertuzumab, which hampers the effective application of these two antibodies.

Several mechanisms have been proposed to explain the observed synergism of trastuzumab and pertuzumab in treating HER2-positive cancers, including breast cancer, ovarian cancer, non-small cell lung cancer, and gastric cancer [178,179,180,181]. These mechanisms include: (1) the synergism due to the different functions of these two antibodies in targeting HER2-positive cancer cells (reviewed in [182]); (2) synergism due to composition-independent inhibitory effects of the combination of the two antibodies in a wide range of HER2/HER3 composition [183]; and (3) synergism of trastuzumab and pertuzumab partly due to the enhanced binding affinity towards the HER2 molecule that originated from the cooperative interactions between the two antibodies [184]. While the first one is supported by some experimental data, the other two mechanisms are purely based on computational models. Thus, the mechanism one will be discussed in more detail.

**Figure 3:**
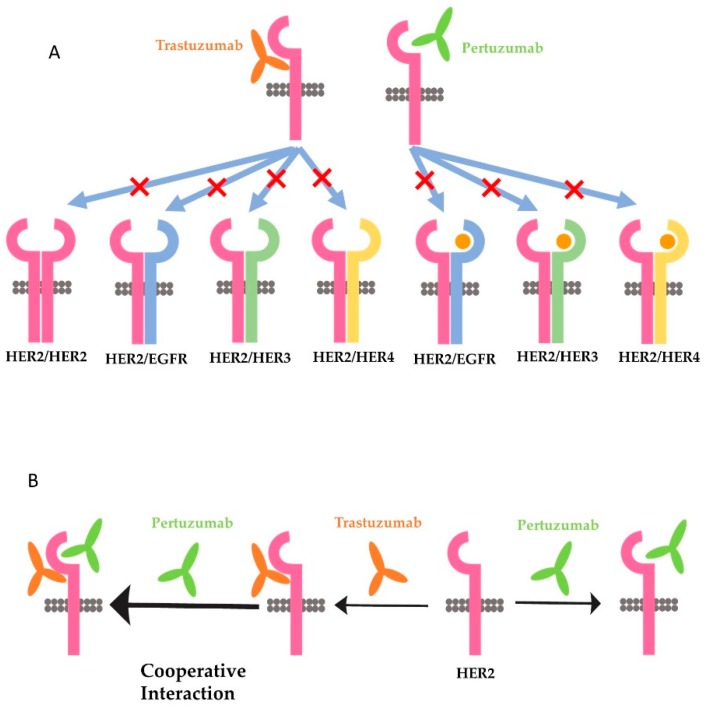
Proposed models illustrating the synergism of trastuzumab and pertuzumab. (**A**) A favored theory by assuming the distinctive action of trastuzumab and pertuzumab on HER2 homo- and heterodimers with or without ligand. (**B**) Hypothesis based on computational analysis, which suggests cooperative interactions between the two antibodies.

Many different functions have been attributed to trastuzumab and pertuzumab, and all of them could be used to explain the synergism of the two antibodies. One favored theory is that trastuzumab inhibits the homodimerization of HER2 and the downstream signaling pathways activated by HER2 homodimer, whereas pertuzumab preferentially blocks the heterodimerization of HER2 with EGFR, HER3, and HER4, and the downstream signaling pathways activated by HER2 heterodimers (Figure 3A) [182,185]. This theory is supported by some research data. It was reported that trastuzumab, not pertuzumab, disrupts ligand-independent signaling mediated by the HER2 homodimer [186]. It was also shown that trastuzumab inhibited ligand-independent HER2 and HER3 interaction [187]. On the other hand, pertuzumab prevents ligand-induced dimerization of HER2 with HER3 [171,174,186,188].

However, several issues need to be resolved to sustain this theory. First, what is the structure basis for pertuzumab to only inhibit HER2 heterodimerization but not HER2 homodimerization? There is no data to indicate that HER2 homodimerization is not mediated by the dimerization domain. Conversely, a recent study with crystallized HER2 ECD homodimer indicates that HER2 homodimer is formed through the interaction of domain II in one protomer with the C-shaped pocket created by domain I–III of the adjacent HER2 protomer [189]. The same research further showed that pertuzumab, but not trastuzumab, blocks the homodimerization of HER2 [189]. In our recent study with Chinese hamster ovary (CHO) cells stably expressing only HER2 (no other HER receptors), HER2 massively forms homodimers due to overexpression. However, trastuzumab does not inhibit the formation of HER2 homodimers [104]. In addition, trastuzumab does not block the phosphorylation of any of the major C-terminal tyrosine residues. However, trastuzumab stimulates strong ADCC in the cells [104]. Thus, it is likely that pertuzumab, rather than trastuzumab, also inhibits the homodimerization of HER2. Secondly, the findings by Junttila et al. [187] suggest that the ligand-independent HER2/HER3 complex in trastuzumab-sensitive cells is structurally distinct form heregulin-induced HER2/HER3 heterodimers. However, until now no further research to compare the structure of ligand-independent HER2/HER3 heterodimer with the structure of heregulin-induced HER2/HER3 heterodimers has been undertaken.

Despite the problems with the favored theory discussed above, the concept that the synergy is due to the different functions of these two antibodies in targeting HER2-positive cancer cells could still prevail, as increasing amounts of data point to the different functions of these two antibodies in targeting HER2-positive cancer cells. It is likely that pertuzumab acts to disrupt the canonical cell-signaling cascades mediated by activated HER2, especially the signaling pathways activated by heregulin-induced heterodimerization of HER2/HER3. Indeed, so far, no data disputes the role of pertuzumab in inhibiting the formation of HER2 heterodimer and blocking the intracellular signaling downstream of HER2 heterodimers. Instead, more data support the role of pertuzumab in disrupting heregulin-induced HER2/HER3 heterodimers, and the activation of the PI3K/Akt pathway downstream of HER2/HER3 heterodimers [174,186,188]. On the other hand, the action of trastuzumab may come through non-canonical pathways downstream of HER2. Trastuzumab has consistently been shown to elicit strong ADCC [96,97,98,99,100,101,102,103,104]. Trastuzumab is reported to block HER2 endocytosis/downregulation [120,121,122,123], DNA repair [137], proteolytic cleavage of HER2 extracellular domain [126], and angiogenesis [139,140]. It was recently reported that trastuzumab, but not pertuzumab, inhibits autophagy and increases the production of reactive oxygen species in human cardiomyocytes by dysregulating HER2 signaling [190].

The second hypothesis is through computational analysis. This analysis is based on several previous publications [171,180,186,187] that formed bases of Hypothesis 1 [183]. The computational model revealed that trastuzumab and pertuzumab alone or in combination differentially suppress HER receptor-mediated cell signaling. This suppression is dependent on the expression pattern of various HER receptors. Trastuzumab treatment upregulates HER3, which reprograms HER receptor kinetics from HER2 homodimerization to HER2/HER3 heterodimerization. As trastuzumab is more effective in targeting HER2 homodimers and pertuzumab is more effective in targeting HER2/HER3 heterodimers, the synergy is due to the effectiveness of the combination of the two antibodies on targeting both HER2 and HER2/HER3 heterodimers [183]. This hypothesis could be considered to be the extension of Hypothesis 1.

The third hypothesis is also based on a molecular modeling study. This hypothesis suggests that the synergy of trastuzumab and pertuzumab is partly due to the enhanced affinity that originates from the cooperative interactions between the two antibodies (Figure 3B). This model assumes that the two antibodies can colocalize on the extracellular domain of the same HER2 molecule [184]. Following the binding of trastuzumab, the receptor becomes highly plastic, especially on domains I and III, which promotes the association of pertuzumab with HER2. On the other hand, the binding of pertuzumab to HER2 induces novel interactions between HER2 and trastuzumab. The enhanced binding of both antibodies to HER2 inhibits HER2 dimerization and possibly higher oligomerizations with other HER receptor molecules [184]. Since this, it has been reported that the binding of radio-labeled trastuzumab and pertuzumab to HER2-positive cells is increased when both antibodies are applied together compared to when they are applied separately [191]. However, this mechanism was later disputed by another study with experimental characterization by measuring the binding kinetics of trastuzumab and pertuzumab, either the whole antibodies or the F(ab)s, to HER2 extracellular domains [126]. This later research indicates that both pertuzumab and trastuzumab do colocalize on to the same HER2 molecule, but do not augment the binding of each other [192].

## 8. Other Anti-HER2 Strategies beyond Pertuzumab

In addition to trastuzumab and pertuzumab discussed above, many other agents and strategies have also been developed to target HER2. We have discussed the combination of targeting HER2 and PI3K-Akt-mTor above. The other agents and strategies developed simultaneously include the anti-HER2 agents that target the signaling pathways downstream of HER2 include the tyrosine kinase inhibitors (TKIs) such as neratinib and lapatinib, and the antibody-drug conjugate trastuzumab emtansine (T-DM1) [23,176,193,194]. The recent trends and development in this field include the immunotherapy agents such as anti-PD-L1 agent pembrolizumab [195], the bispecific antibodies such as MCLA-128, which targets both HER2 and HER3 [196], and ZW25, which targets different epitopes on the HER2 extracellular domain, the antibody-drug conjugates (ADCs) such as SYD-0985 and DS-8201a, and new anti-HER2 antibodies and pan-HER TKIs [177]. These new agents and novel strategies have resulted in a multitude of opportunities to capitalize on the biology of HER2-positive breast cancer and ultimately improve responses to HER2-targeted therapy.

## 9. Conclusions

**Figure 4:**
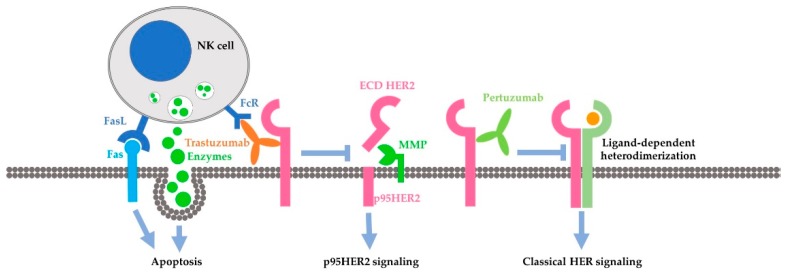
Model proposed in this review by including more recent data. Pertuzumab acts to inhibit the classical HER2-mediated cell-signaling cascades by blocking HER2 dimerization. Trastuzumab acts through ADCC and the inhibition of HER2 cleavage.

The approval of trastuzumab in treating breast cancer changes the paradigm of breast cancer treatment. To increase the effectiveness and overcome the resistance, pertuzumab was later introduced to treat breast cancer patients together with trastuzumab and docetaxel. While a better outcome is achieved by the combination of pertuzumab, trastuzumab and docetaxel, the percentage of HER2-positive breast cancer patients who benefited from the treatment is still low. Understanding the mechanisms of the action and synergy of trastuzumab and pertuzumab is essential to develop novel therapies for increased effectiveness. While more research is needed to resolve the controversy and elucidate the detail of the mechanism, accumulated data have suggested a likely model for the synergy of trastuzumab and pertuzumab. In this model, the synergy is mostly due to the distinctive mode of action of these two antibodies (Figure 4). Pertuzumab may mostly act to inhibit the classical signaling pathways stimulated by active HER2, including receptor dimerization, receptor phosphorylation and the activation of signaling proteins downstream from HER receptors, including Erk and Akt. Furthermore, trastuzumab may mostly act through pathways other than the classical HER2-signaling cascades. Trastuzumab stimulates strong ADCC, and blocks the generation of active p95HER2 fragments by inhibiting the cleavage of HER2, and others. Certainly, further research is needed to completely elucidate the molecular mechanisms underlying the action and synergy of trastuzumab and pertuzumab.
